# Adolescents’ Use of Care for Behavioral and Emotional Problems: Types, Trends, and Determinants

**DOI:** 10.1371/journal.pone.0093526

**Published:** 2014-04-03

**Authors:** Sijmen A. Reijneveld, P. Auke Wiegersma, Johan Ormel, Frank C. Verhulst, Wilma A. M. Vollebergh, Danielle E. M. C. Jansen

**Affiliations:** 1 University Medical Center Groningen, University of Groningen, Department of Health Sciences, Groningen, the Netherlands; 2 University Medical Center Groningen, University of Groningen, Interdisciplinary Center Psychopathology and Emotion Regulation, Groningen, the Netherlands; 3 Erasmus Medical Center, Department of Child and Adolescent Psychiatry, Rotterdam, the Netherlands; 4 Department of Social Sciences, University of Utrecht, Utrecht, the Netherlands; Langeveld Institute, Utrecht University, Netherlands

## Abstract

**Objective:**

While adolescents use various types of care for behavioral and emotional problems, evidence on age trends and determinants per type is scarce. We aimed to assess use of care by adolescents because of behavioral and emotional problems, overall and by type, and its determinants, for ages 10–19 years.

**Methods:**

We obtained longitudinal data on 2,230 adolescents during ages 10–19 from four measurements regarding use of general care and specialized care (youth social care and mental healthcare) in the preceding 6 months, the Child Behavior Checklist (CBCL) and Youth Self-Report, and child and family characteristics. We analyzed data by multilevel logistic regression.

**Results:**

Overall rates of use increased from 20.1% at age 10/11 to 32.2% at age 19: general care was used most. At age 10/11 use was higher among boys, at age 19 among girls. Use of general care increased for both genders, whereas use of specialized care increased among girls but decreased among boys. This differential change was associated with CBCL externalizing and internalizing problems, school problems, family socioeconomic status, and parental divorce. Preceding CBCL problems predicted more use: most for mental health care and least for general care. Moreover, general care was used more frequently by low and medium socioeconomic status families, with odds ratios (95%-confidence intervals): 1.52 (1.23;1.88) and 1.40 (1.17;1.67); youth social care in case of parental divorce, 2.07 (1.36;3.17); and of special education, 2.66 (1.78;3.95); and mental healthcare in case of special education, 2.66 (1.60;4.51).

**Discussion:**

Adolescents with behavioral and emotional problems use general care most frequently. Overall use increases with age. Determinants of use vary per type.

## Introduction

Estimates of rates of behavioral and emotional problems among adolescents vary from 10 to 25%. These problems may lead to restrictions in their daily functioning and to severe long-term effects. [1]–[4] Ideally, all adolescents with such impairing problems should receive appropriate, evidence-based interventions. In reality, only a minority of them will receive care, [1], [5]–[8] that is, either general or specialist care. Specialist care involves either youth mental healthcare, or youth social care. [5], [6] In practice, various factors hamper proper use of adequate care.

Use of care has been shown to be more likely in case of more severe problems and associated impairments, [5]–[7], [9], [10] and to depend further on factors such as child age and gender, [5], [6] parental [5]–[7] and teacher perceptions of problems, [6], [11] and environmental factors, [5], [7], [11] like not living with two biological parents and educational difficulties of the child. [9] However, evidence concerning the influence of these determinants is inconclusive. This may be due to differences in the types of care that were studied, in the factors that were assessed, and in the ages of the adolescents involved. [5], [6] For example, Ford and co-authors found that in the age range 5–15 years socioeconomically disadvantaged children were more likely to receive youth social care, while boys were more likely to receive mental healthcare. [6] Use of any care for behavioral and emotional problems has also been shown to vary by age. [5], [6] This may be due to an age-specific effect of determinants. For example, a study of Ford and co-authors also shows that determinants of use of primary care varies by age, with only child behavioral problems playing a role at ages 5–10, whereas more factors contributed at ages 11–15. [6].

A theoretical model may help to structure the role of various factors in the seeking and obtaining of care for behavioral and emotional problems. An useful and widely used model is Andersen’s socio-behavioral model of care utilization. [7], [11], [12] This model assumes that use of care is subject to factors that can be either predisposing, enabling, or expressing need. Predisposing factors refer to everything that might prompt seeking and using care, e.g. educational level. Enabling factors involve the means by which services might be accessed, e.g. the competences to achieve aims. Need factors in this study include health problems and the degree to which they require care, e.g. the severity of the health problem concerned. This model may be applied to specific services or to the entire care system. [7] Need factors should probably be included in any prediction of use of care for behavioral and emotional problems, given the wealth of data on the association between perceived behavioral and emotional problems and use of care. [5]–[7], [9] Determinants of use are likely to vary by type of care, as use of care is the result of various care seeking processes. [13] This has first to do with the severity of the problems. Typically, less severe problems can be expected to be handled in primary care, according to Goldberg and Huxley’s model of filters of care which presumes a number of filters to be passed before a person reaches more complex and intensive types of care. [5], [13] Second, mental health care traditionally aims at children with mental problems, i.e. regarding behavior or emotion. In contrast, youth social care traditionally aims at supporting the social and economic context of youth. [5], [7], [11] Because of these different aims, behavioral and emotional problems can be expected to be associated most strongly with use of mental health care, whereas factors like parental divorce and poverty can be expected to be associated with youth social care. However, circumstances leading to use of care may be interconnected as well. For instance, social circumstances like parental divorce and unemployment, [9] or living in a deprived area, [14] may lead to behavioral and emotional problems. Evidence on the factors associated with use of various types of care is very scarce.

The aim of this study is first to assess by gender and age the use of care by adolescents in the age range 10–19 years because of behavioral and emotional problems. A second aim is to assess the factors determining this use overall and by type of care, along with the degree to which these factors explain changes in use by age. Andersen’s socio-behavioral model of health care utilization will be used as the theoretical basis for this study.

## Methods

### Sample and Procedure

The study was performed as part of the TRacking Adolescents’ Individual Lives Survey (TRAILS) study, a large prospective population study of Dutch adolescents, designed to examine and explain the progress of mental health and social development from pre-adolescence into adulthood. [15]Enrolment for TRAILS started in 2001. Children were randomly selected from the Population Registers of five municipalities in the north of the Netherlands and were included if they were aged 10–11 and attended a school that was willing and able to participate: *N = *2,935 children. Of these, 2,230 provided informed consent to participate from both parent and child (76.0%; T1). Non-respondents more frequently were boy, or single child, or had a low-educated parent, but they did not differ regarding psychopathology. [4], [7], [10] The present study involves data from the first four measurement waves of TRAILS (T1–T4), running from 2001 until 2010. Mean age at T1 was 11.1 years; standard deviation 0.55. At T2, T3, and T4 mean ages were 13.6, 16.2, and 19.1 years, respectively.

During the first and third measurement wave, well-trained interviewers visited one of the parents or their guardian at their homes to administer an interview covering the child’s developmental history and somatic health, parental psychopathology, and care utilization. The parent or guardian was also asked to fill out a questionnaire at each wave, as was the adolescent. [16] The design of each wave of the study was separately approved by the Dutch National Medical Ethics Committee (www.ccmo.nl), including the written informed consent by both child and parents.

### Measurements

The data concerned service use, and predisposing, enabling, and need factors, based on Andersen’s model; for each factor, the wave at which it was measured is indicated as “(T1),” etc.

*Service use* was measured as parent-reported use of any type of professional care because of behavior or emotional issues of the child in the past six months (T1–4). In the Dutch care system, children and adolescents with behavioral and emotional problems can contact either preventive child healthcare, their family physician or the office for youth care. If more specialized care is needed, these can refer either to youth mental health care or to youth social care. Youth mental health care comprises care provided by child psychologists, and child and adolescent psychiatrists. Youth social care comprises care provided by youth and social workers and includes child protection. All types of care requiring referral have been included under the heading “specialized care”. Based on this structure of the care system, care was categorized as general care (preventive child healthcare, family physician, home care, etc.), youth social care (youth social work, youth protection, etc.), and youth mental healthcare (child psychologist, psychiatrist, etc.). The latter two were also denoted as “specialized care.” [4].

*Predisposing factors* refer to everything that might predispose an adolescent to seek and use a specific service. In this study, the predisposing factors include age (T1–4), gender (T1), degree of urbanization (T1), family structure (T1–2), ethnicity (T1), educational level of adolescent (T1–2) and parents (T1), and socioeconomic status of the parent(s) (T1).

*Urbanization* was assessed by the number of residential addresses per 3.14 square kilometers (i.e., by drawing a circle with a radius of one kilometer from each point). [7], [8] Following the guidelines of Statistics Netherlands, it was dichotomized as less than 1000 for rural and 1000 and more for urban (http://www.rivm.nl/vtv/object_map/o2617n21780.html).

*Family structure* was measured as not having continuously lived with two parents from birth, denoted as “one biological parent.” If yes, this was due to divorce in 90% of cases (remainder almost always single mothers by choice). Of those with one biological parent, one-third had a stepparent at T1.

*Educational level* of the adolescent was measured by parent report in two ways. The first one concerned progress in primary education at T1, measured as: 1) regular primary education, 2) special primary education, 3) having repeated one or two grades, and 4) having skipped a grade. The second one concerned school level at T2, being higher secondary school, lower secondary or vocational school, or primary school or special education. For standard pupils, the transition from primary to secondary school occurs around age 12 in the Dutch system.

*Parental education* consisted of five levels: 1) elementary education, 2) lower tracks of secondary education, 3) higher tracks of secondary education, 4) senior vocational education, and 5) university education.

*Parental occupational level* was measured based on the International Standard Classification for Occupations, [15] as low, medium, or high.

Finally, *family socioeconomic status* was computed as the aggregate of parental occupational level, parental educational level, and family income: Cronbach alpha: 0.84. This was categorized as low (lowest 25%), medium (next 50%) and high (highest 25%). [17].

*Enabling factors* are related to the means by which adolescents might access mental healthcare. The enabling factors considered in this study were perceived social support of children from parents and peers, and perceived self-competence (all T1). “Perceived social support” was measured as perceived affection from, and behavioral confirmation toward both parents and classmates, respectively. [18] *Perceived self-competence* was measured by the Self-Perception Profile for Children (SPPC) questionnaire, which measures self-reported competencies in youths. Previous research has shown it to be highly valid and reliable in the Dutch setting; [19] Cronbach’s alphas in this study varied from 0.58 (SPPC Sports) to 0.81 (SPPC Appearance).

*Need factors* concerned how adolescents viewed their own mental health and how their parents perceived need. These views were assessed using the Youth Self-Report (YSR) and the Child Behavior Checklist (CBCL) with a time-frame of the past 6 months, for waves 1–3. The YSR and CBCL are highly reliable and valid measurements of behavioral and emotional problems over the preceding six months. [20] They are filled out by parents and adolescents or pre-adolescents, respectively, but in other respects contain similar items. We used age-standardized scores on two broad-band dimensions: internalizing (anxious/depressed, withdrawn/depressed, and somatic complaints) and externalizing problems (aggressive behavior and rule-breaking behavior). Cronbach’s alphas for the first wave of this study were: YSR internalizing –0.87; YSR externalizing –0.85; CBCL internalizing –0.85; CBCL externalizing - 0.90.

### Analysis

We first imputed missing data on the CBCL and YSR based on the multivariate normal model, [21], [22] as implemented in the NORM software. This procedure minimizes the loss of statistical power, provides correctly estimated standard errors, and preserves the characteristics of the data set as a whole. [23] Adolescents with missing values in categorical independent variables were retained in the analyses by creating separate dummies per variable. Next, we computed descriptives of the background of the adolescents concerned, overall and by gender. Third, we tested whether rates of the use of various services differed by age, using chi-square tests. Fourth, we assessed which factors determined use of various services and whether these differed by age, using logistic regression analyses. We first did this for blocks of determinants, predisposing and enabling factors (i.e., sociodemographic ones) and need factors (levels of behavioral and emotional problems), and then for all determinants combined. Regarding need factors, we examined the associations with current needs, that is, current CBCL and YSR scores (for use in waves 1–3), and with previous needs, that is, CBCL and YSR scores during the preceding assessment (for use in waves 2–4). Finally, we examined whether the effect of level of problems on trends in use of services differed for internalizing as compared to externalizing problems and whether these effects varied by gender, again using logistic regression.

We performed the first until third step using IBM SPSS 20.0 (/www01.ibm.com/software/analytics/spss/). The fourth and fifth steps were done with multilevel techniques in MLwiN 2.22 (www.cmm.bristol.ac.uk/MLwiN), with the highest level being the adolescent with his/her personal and family characteristics, and the lower level being each measurement wave that was available for that adolescent. In that way, we could account for the fact that various measurements for the same adolescent were correlated. [3], [7], [24] Moreover, if an outcome was not available for one wave, the adolescent still contributed to the modeling for the other waves.

In the multilevel models, the probability of the response of the measurement at the i-th wave in the j-th individual was modelled as follows, following various previous report, e.g. [15], [25] ^,^ [15] ^,^ [26]:
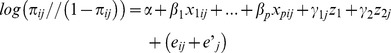
where α represents the constant term;

β_1_ … β_p_ represent the regression coefficients of the wave-specific explanatory variables x_1_ … x_p_;

γ_1_ and γ_2_ represent the regression coefficients of the individual explanatory variables z_1_ and z_2_;

e_ij_ represents the wave-level residuals, and e’_j_ represents the individual-level residuals. These residuals are also denoted as random variables, with a zero expectation, and σ_ij_^2^ and σ_i_^2^ as respective variances. Random variation at wave level was assumed to be approximately binomially distributed, based on the dichotomized outcomes. [27] We did not assess covariance terms between wave- and individual-level. Models were fitted using the most accurate procedure available, i.e. a predictive quasi-likelihood procedure in combination with a second order Taylor expansion series. [28]

## Results

Table 1 provides descriptives of the background of the adolescents concerned. At baseline (ages 10/11), they had a predominantly Dutch-born ethnic background (89%), most lived in a two-parent family (76%), and in a rural or semi-rural area (72%), and most had a regular progress at primary school (75%).

**Table 1:** Background characteristics of the adolescents who reported on use of care at T1, by gender*.

	Girls	Boys	Total
	(n = 1076)	(n = 1024)	(n = 2100)
SES family			
Low	259 (23.2%)	294 (27.4%)	553 (25.3%)
medium	585 (52.5%)	499 (46.5%)	1084 (49.5%)
High	271 (24.3%)	280 (26.1%)	551 (25.2%)
One-parent family (T1)	274 (24.2%)	255 (23.2%)	529 (23.7%)
Parental divorce 2< years ago (T2)	62 (5.8%)	55 (5.4%)	117 (5.6%)
Non-Dutch ethnicity	123 (10.9%)	114 (10.4%)	237 (10.6%)
Urban (>1000 addresses/km2)	309 (27.7%)	296 (27.3%)	605 (27.5%)
Progress at school (T1)			
Regular	895 (79.1%)	786 (71.6%)	1681 (75.4%)
special education	40 (3.5%)	84 (7.7%)	124 (5.6%)
repeated 1 or 2	172 (15.2%)	205 (18.7%)	377 (16.9%)
skipped class	25 (2.2%)	23 (2.1%)	48 (2.2%)
Child school level at T2			
primary school/special education	111 (9.9%)	158 (14.4%)	270 (12.1%)
lower/vocational secondary	625 (55.2%)	592 (53.9%)	1217 (54.6%)
higher secondary	395 (34.9%)	348 (31.7%)	743 (33.3%)
CBCL Externalizing (T1)#	7.4 (6.2)	9.7 (7.4)	8.5 (6.9)
CBCL Externalizing (T2)#	5.5 (6.0)	6.4 (6.8)	6.0 (6.4)
CBCL Externalizing (T3)#	5.7(6.0)	6.0 (6.6)	5.8 (6.3)
CBCL Internalizing (T1)#	8.0 (6.0)	7.8 (5.9)	7.9 (6.0)
CBCL Internalizing (T2)#	6.6 (5.8)	5.9 (5.7)	6.3 (5.8)
CBCL Internalizing (T3)#	6.7 (6.5)	5.2 (5.3)	6.0 (6.0)
YSR Externalizing (T1)#	7.6 (5.3)	9.8 (6.6)	8.7 (6.1)
YSR Externalizing (T2)#	8.6 (6.0)	9.4 (6.5)	9.0 (6.2)
YSR Externalizing (T3)#	9.2 (6.6)	10.2 (6.9)	9.7 (6.8)
YSR Internalizing (T1)#	12.1 (7.4)	10.5 (7.1)	11.3 (7.3)
YSR Internalizing (T2)#	11.9 (7.8)	8.1 (6.3)	10.0 (7.4)
YSR Internalizing (T3)#	11.7 (7.9)	6.9 (5.9)	9.5 (7.5)
At least once use of care because of behavioral/emotional problems (T1– T4)	639 (56.4%)	550 (50.1%)	1189 (53.3%)

*Numbers do not always add up to the total sample due to missing values.

# Cut-offs for clinical scores for Dutch adolescents (ages 12–18 years) are, for girls/boys: CBCL Externalizing –16/19; CBC Internalizing −15/14; YSR Externalizing; YSR Externalizing −21/20; YSR Internalizing 24/18.

All cut-offs refer to the lowest raw score included in ‘clinical’. [30].

Table 2 shows that at all ages, use of general care was higher than of specialized care. Overall rates of use increased from 20.1% at age 10/11 to 32.2%. at age 19. At age 10/11 use was higher among boys than among girls, but towards age 19, it had more than doubled among girls while being rather stable among boys. This gender-differential increase implies that rates at age 10/11 years are higher for boys, but at age 19 much higher for girls. Looking at types of care, the increases for girls occurred in both general care and specialized care, whereas for boys an increase only occurred for use of general care. Use of specialized (youth social and mental health) care in boys decreased. Moreover, quite a few adolescents used several types of care within the assessment period, all prevalences of use per age-category adding to over 100%.

**Table 2:** Use of various types of care because of behavioral and emotional problems in the past six months, by gender and measurement wave (mean age per wave in years): numbers and percentages of users, p-values for gender differences, and p-values for trend across waves.

	T1 (11.1 yrs)		T2 (13.6 yrs)		T3 (16.2 yrs)		T4 (19.1 yrs)		
	N	%	n	%	n	%	N	%	Trend#
Any care$									
-girls	192	17.8	274	28.0	277	36.9	327	38.5	<0.001
-boys	231	22.6	285	30.4	164	25.2	205	25.6	0.41
-total	423	20.1**	559	29.2	441	31.5 ***	532	32.2 ***	<0.001
General care									
-girls	151	14.0	229	23.4	247	32.9	290	34.1	<0.001
-boys	142	13.9	224	23.9	123	18.9	167	20.8	0.03
-total	293	14.0	453	23.6	370	26.4***	457	27.7 ***	<0.001
Youth social care									
-girls	51	4.7	72	7.3	55	7.3	54	6.4	0.14
-boys	80	7.8	80	8.5	40	6.1	57	7.1	0.29
-total	131	6.2**	152	7.9	95	6.8	111	6.7	0.81
Mental healthcare									
-girls	23	2.1	30	3.1	43	5.7	68	8.0	<0.001
-boys	66	6.4	67	7.2	35	5.4	54	6.7	0.86
-total	89	4.2***	97	5.1***	78	5.6	122	7.4	<0.001
*Any specialized care*									
* -girls*	*67*	*6.2*	*88*	*9.0*	*85*	*11.3*	*101*	*11.9*	*<0.001*
* -boys*	*130*	*12.7*	*117*	*12.5*	*61*	*9.4*	*84*	*10.5*	*0.047*
* -total*	*197*	9.4***	*205*	10.7**	*146*	*10.4*	*185*	*11.2*	*0.093*
Nr of respondents									
-girls	1076		980		751		850		
-boys	1024		937		651		801		
-total	2100		1917		1402		1651		

P-values for gender differences based on chi-square tests: *p<0.05; **p<.01; ***p<0.001.

# P-values for linear trend across waves based on chi-square tests.

$ The summation of numbers per type exceed the numbers mentioned for ‘Any care’ because some adolescents used more than one type of care. The same holds for ‘Any specialized care’: some adolescents used both youth social care, and mental healthcare.

Multilevel logistic regression showed that age trends in overall use indeed differ by gender, as shown by statistically significant interactions of gender with wave (Table 3). Adjusted for gender and wave, several predisposing enabling and need factors were associated with overall higher use of any care. Mutual adjustment between factors decreased the strength of most of these associations, as shown by generally lower odds ratios, OR (Table 3, most right columns). In these models, we separately adjusted for CBCL and YSR scores in either the concurrent or the preceding wave. OR for the former were mostly somewhat higher than for the latter, though they were always in the same direction; the latter are not shown. Moreover, associations with higher use were stronger for the parent-reported CBCL scales than for the adolescent-reported YSR scales. Associations of need factors with higher use of care decreased relatively strongly after mutual adjustment, indicating their relatively high correlations.

**Table 3:** Determinants of use of care, adjusted for gender and wave, and additionally for all shown factors: odds ratios (OR), and 95% confidence intervals (CI) from multilevel logistic regression.

	Gender/wave adjusted			Fully adjusted		
	OR	*95% CI*		OR	*95% CI*	
**Background**						
Female (vs. male)	**0.74**	***0.60***	***0.93***	0.79	*0.62*	*1.01*
Wave 1 (11.1 years)	1			1		
“ 2 (13.6 years)	**1.51**	***1.23***	***1.84***	**1.75**	***1.40***	***2.19***
“ 3 (16.2 years)	1.17	*0.93*	*1.47*	**1.49**	***1.16***	***1.92***
“ 4 (19.1 years)	1.20	*0.96*	*1.49*	–		
Female * wave 1	1			1		
“ * wave 2	1.19	*0.89*	*1.60*	1.06	*0.77*	*1.45*
“ * wave 3	**2.34**	***1.70***	***3.22***	**1.97**	***1.40***	***2.78***
“ * wave 4	**2.45**	***1.81***	***3.31***	–		
**Predisposing factors**						
SES of family (T1) (reference is high)						
low	**1.64**	***1.37***	***1.96***	1.15	*0.91*	*1.44*
Medium	**1.52**	***1.31***	***1.76***	**1.25**	***1.04***	***1.49***
One-parent family (T1) yes (vs. no)	**1.73**	***1.49***	***2.00***	**1.34**	***1.12***	***1.60***
Rec. par. divorce (T2) yes (vs. no)	**1.59**	***1.21***	***2.09***	1.33	*0.98*	*1.82*
Non-Dutch ethnicity (T1) yes (vs. no)	0.92	*0.73*	*1.15*	**0.74**	***0.57***	***0.98***
Rural (T1) (vs. urban, >1000)	**1.16**	***1.01***	***1.34***	1.08	*0.92*	*1.27*
Progress at school (T1) (reference is regular)						
special education	**2.04**	***1.54***	***2.69***	1.31	*0.91*	*1.88*
repeated 1 or 2	**1.37**	***1.16***	***1.61***	1.08	*0.87*	*1.35*
skipped class	0.75	*0.48*	*1.18*	0.82	*0.48*	*1.40*
Child school level (T2) (ref. is higher secondary)						
primary/special	**2.05**	***1.66***	***2.52***	**1.42**	***1.05***	***1.93***
lower/vocational secondary	**1.46**	***1.27***	***1.67***	**1.27**	***1.06***	***1.51***
**Enabling factors**						
Social support father (T1)	1.13	*0.98*	*1.30*	1.02	*0.84*	*1.25*
Social support mother (T1)	**1.18**	***1.02***	***1.37***	1.05	*0.85*	*1.29*
Social support friends (T1)	0.96	*0.84*	*1.09*	0.87	*0.74*	*1.02*
Self-competence learning (T1)	1.06	*0.93*	*1.20*	0.89	*0.76*	*1.04*
Self-competence friends (T1)	**1.35**	***1.19***	***1.54***	1.11	*0.95*	*1.31*
Self-competence sport (T1)	1.04	*0.92*	*1.18*	0.99	*0.85*	*1.16*
Self-competence appearance (T1)	**1.18**	***1.03***	***1.35***	1.00	*0.83*	*1.19*
Self-competence behavior (T1)	**1.29**	***1.14***	***1.47***	1.09	*0.93*	*1.28*
Self-competence general (T1)	**1.29**	***1.11***	***1.50***	0.95	*0.77*	*1.18*
**Need factors**						
CBCL Externalizing, current (T1–T3)	**1.55**	***1.46***	***1.65***	**1.29**	***1.19***	***1.41***
CBCL Internalizing, current (T1–T3)	**1.56**	***1.46***	***1.66***	**1.28**	***1.18***	***1.39***
YSR Externalizing, current (T1–T3)	**1.58**	***1.36***	***1.84***	0.99	*0.91*	*1.08*
YSR Internalizing, current (T1–T3)	**1.27**	***1.19***	***1.36***	**1.11**	***1.02***	***1.21***
CBCL Externalizing, preceding (T2–T4)#	**1.37**	***1.28***	***1.46***	–		
CBCL Internalizing, preceding (T2–T4)#	**1.32**	***1.23***	***1.40***	–		
YSR Externalizing, preceding (T2–T4)#	**1.15**	***1.08***	***1.23***	–		
YSR Internalizing, preceding (T2–T4)#	**1.19**	***1.11***	***1.27***	–		

Bold = p<. 05.

# measurement of need at preceding wave was used as predictor for use in next wave, e.g. CBCL at T1 was used as predictor of use at T2, etc.

The adjustment for predisposing, enabling, and need factors had some influence on the associations of use of any care with gender, measurement wave, and their interaction. In particular, the OR for waves increased somewhat, whereas the OR for the interaction of gender and wave decreased. Thus, after this adjustment, increases by wave in use of any service by age were more similar for boys and girls. The effect of need did not vary by gender.

In its top rows Table 4 shows the results as presented in Table 3, but now also per type of care. Interactions of gender and wave were found for all types of care, except for youth social care at wave 4. Model 2 in Table 4 shows that adjustment for all factors increased ORs somewhat for wave but decreased them for the gender-wave interaction. This again indicates that these factors do not account for the overall increase in use at increasing ages, but that they account for some of the differential increase in use of care between girls and boys with increasing age. The latter effect was particularly large for use of youth social care.

**Table 4:** Determinants of use of care in three models including: 1) only gender and wave; 2) all factors; and 3) factors derived from stepwise forward selection.

	Any care			General care			Youth social care			Mental health care		
	OR	*95% CI*		OR	*95% CI*		OR	*95% CI*		OR	*95% CI*	
**1) Gender/wave model (contains gender, wave and their interaction)**												
Female (vs. male)	**0.74**	***0.60***	***0.93***	1.01	*0.78*	*1.30*	**0.59**	***0.40***	***0.85***	**0.32**	***0.19***	***0.53***
Wave 1 (11.1 yrs; reference)	1			1			1			1		
“ 2 (13.6 years)	**1.51**	***1.23***	***1.84***	**1.95**	***1.55***	***2.47***	1.11	*0.80*	*1.53*	1.13	*0.79*	*1.61*
“ 3 (16.2 years)	1.17	*0.93*	*1.47*	**1.45**	***1.11***	***1.89***	0.81	*0.55*	*1.20*	0.83	*0.54*	*1.28*
“ 4 (19.1 years)	1.20	*0.96*	*1.49*	**1.65**	***1.29***	***2.11***	**0.49**	***0.28***	***0.86***	1.06	*0.73*	*1.55*
Female * wave 1 (reference)	1			1						1		
“ * wave 2	1.19	*0.89*	*1.60*	0.96	*0.69*	*1.33*	1.45	*0.89*	*2.37*	1.31	*0.68*	*2.52*
“ * wave 3	**2.34**	***1.70***	***3.22***	**2.09**	***1.47***	***2.97***	**2.02**	***1.16***	***3.52***	**3.45**	***1.76***	***6.76***
“ * wave 4	**2.45**	***1.81***	***3.31***	**1.95**	***1.40***	***2.72***	1.50	*0.88*	*2.54*	**3.82**	***2.07***	***7.05***
**2) Fully adjusted model (contains all variables as shown in ** **Table 3** **, but ORs for those variables are not shown)**												
***Gender and Wave ( = age)*** Female (vs. male)	0.79	*0.62*	*1.01*	1.01	*0.77*	*1.33*	0.73	*0.48*	*1.12*	**0.30**	***0.17***	***0.55***
Wave 1 (11.1 yrs; reference)	1			1			1			1		
“ 2 (13.6 years)	**1.75**	***1.40***	***2.19***	**2.17**	***1.70***	***2.77***	1.30	*0.90*	*1.89*	1.31	*0.87*	*1.96*
“ 3 (16.2 years)	**1.49**	***1.16***	***1.92***	**1.72**	***1.30***	***2.28***	1.09	*0.69*	*1.71*	1.10	*0.67*	*1.78*
“ 4 (19.1 years)	–			–			–			–		
Female * wave 1 (reference)	1			1			1			1		
“ * wave 2	1.06	*0.77*	*1.45*	0.88	*0.63*	*1.24*	1.26	*0.72*	*2.20*	1.20	*0.57*	*2.54*
“ * wave 3	**1.97**	***1.40***	***2.78***	**1.80**	***1.24***	***2.60***	1.52	*0.81*	*2.87*	**2.68**	***1.22***	***5.85***
“ * wave 4	–			–			–			–		
**3) Stepwise forward model (model containing only the variables as shown)**												
***Gender and wave ( = age)*** Female (vs. male)	0.80	*0.64*	*1.01*	1.03	*0.80*	*1.34*	0.70	*0.47*	*1.04*	**0.34**	***0.20***	***0.59***
Wave 1 (11.1 yrs; reference)	1			1			1			1		
“ 2 (13.6 years)	**1.71**	***1.39***	***2.12***	**2.12**	***1.67***	***2.68***	1.27	*0.90*	*1.80*	1.26	*0.86*	*1.85*
“ 3 (16.2 years)	**1.44**	***1.13***	***1.84***	**1.68**	***1.28***	***2.20***	1.01	*0.66*	*1.55*	1.00	*0.63*	*1.60*
“ 4 (19.1 years)	–			–			–			–		
Female * wave 1 (reference)	1			1			1			1		
“ * wave 2	1.06	*0.78*	*1.43*	0.88	*0.63*	*1.23*	1.23	*0.73*	*2.07*	1.09	*0.55*	*2.19*
“ * wave 3	**1.98**	***1.43***	***2.75***	**1.82**	***1.27***	***2.61***	1.50	*0.82*	*2.74*	**2.53**	***1.22***	***5.85***
“ * wave 4	–			–			–			–		
***Predisposing factors***												
SES of family												
high (reference)	–			1			–			–		
low				**1.52**	***1.23***	***1.88***						
Medium				**1.40**	***1.17***	***1.67***						
Recent parental divorce (T2) yes (vs. no)	**1.45**	***1.08***	***1.95***	–			**2.07**	***1.36***	***3.17***	–		
Progress at school (T1)												
regular (reference)	1			–			1			1		
special education	1.64	***1.21***	***2.22***				**2.66**	***1.78***	***3.95***	**2.69**	***1.60***	***4.51***
repeated 1 or 2	1.20	*1.00*	*1.44*				1.19	*0.88*	*1.62*	**1.76**	***1.22***	***2.54***
skipped class	0.80	*0.48*	*1.33*				0.66	*0.23*	*1.91*	–		
***Need factors***												
CBCL Externalizing, current	**1.29**	***1.19***	***1.39***	**1.19**	***1.10***	***1.30***	**1.41**	***1.26***	***1.59***	**1.61**	***1.40***	***1.86***
CBCL Internalizing, current	**1.29**	***1.19***	***1.40***	**1.15**	***1.05***	***1.25***	**1.61**	***1.43***	***1.81***	**1.41**	***1.22***	***1.63***
YSR Externalizing, current	1.00	*0.92*	*1.08*	0.99	*0.90*	*1.07*	1.02	*0.89*	*1.17*	1.01	*0.85*	*1.19*
YSR Internalizing, current	**1.11**	***1.02***	***1.20***	1.09	*1.00*	*1.18*	1.10	*0.96*	*1.26*	**1.19**	***1.00***	***1.40***

**Bold** denotes p<.05.

‘−’ variable has not been included.

Odds ratios (OR), and 95% confidence intervals (CI) for use of care, derived from multilevel logistic regression models.

The bottom part of Table 4 shows the results of a stepwise forward selection of determining factors according to the Andersen model, again for any care and the three types of care, with need for care always included. Only a limited number of factors were associated with higher use, both for any care and for the three types of care, separately. These particularly concerned the parent-reported need factors (i.e., CBCL scores) and poor progress during primary education (all types except for general care), lower family SES (general care), and recent parental divorce (youth social care). This small set of factors accounts for most of the differences in odds ratios between the gender/wave and the fully adjusted models. The effect of need factors did not vary by gender except for that of adolescent-reported internalizing problems; that effect was slightly larger for boys (OR 1.03; 95% confidence interval 1.00 to 1.07).

## Discussion

We found that in the age range 10–19 years, the use of care services because of behavioral and emotional problems was rather high but mostly involved general care. At age 10/11 use was much higher for boys, but during adolescence use more than doubled for girls, whereas it was rather stable for boys. This led to a much higher use by girls at age 19, with the differential increase being due to specialized care, in particular, youth mental healthcare. Part of this differential change can be accounted for by a limited set of factors: in particular, needs as measured by parent-reported behavioral and emotional problems of the adolescent. In addition, some predisposing factors contribute to this differential change such as family SES for general care, parental divorce, and special education for youth social care, and special education for youth mental healthcare. These factors were all associated with higher use.

Our finding of increasing use by age confirms the available evidence but decidedly shows that the increase strongly differs by gender and by type of care. This especially holds for use of specialized care, which even decreased slightly among boys as they grew older. This differentiation may also explain the heterogeneity in findings up until now, where studies either focused on young [28] or older adolescents, and/or did not adjust for age, gender, and their interaction when assessing the role of other predictors. [6], [10] Re-analyses of these data with inclusion of an age/gender interaction might be of interest.

Needs, measured by CBCL and YSR, were the strongest predictor of higher use of care, with associations being stronger for concurrent than for past needs, and stronger for parent-reported than for adolescent-reported needs. The associations were considerable: adjusted for age/gender, a one standard deviation change in CBCL score was associated with 1.55 higher odds for use of any care. The dominance of parent-report may be interpreted by the rather strong impact parents still have on the help-seeking of their child at these ages and confirms previous findings regarding this. [6] The only exception concerns adolescent-reported internalizing problems which, after mutual adjustment, were still associated with use of youth mental healthcare. An explanation may be that in particular emotional problems affect the adolescent’s well-being and thus stimulate them to seek care. Mental healthcare is then probably more likely to be administered than youth care, since the latter targets the adolescent’s social environment to a relatively greater degree.

We found rather similar associations between parent-reported need factors and use of youth mental health care and of youth social care. One might expect somewhat stronger associations for youth mental health care which specifically targets at mental problems. The fact that this is not the case may be explained in several ways. First, social and economic problems such as parent divorce and unemployment have been shown to lead to adolescent mental health problems. [5]–[7], [9] This would imply that the association of parent-reported behavioral and emotional problems with youth social care is in fact due to the role of underlying social and economic problems. Some support for this explanation is provided by our finding that - in addition to behavioral and emotional problems - parental divorce was also independently associated with use of youth social care but not with that of youth mental health care. A second explanation may be that youth social care and youth mental health care simply serve partially overlapping groups of adolescents. A third explanation might be that need factors are always more important than predisposing and enabling factors regarding use of care in case of behavioral and emotional problems, independent of the type of care. Further research is needed to disentangle these explanations.

Interestingly, the effect of needs hardly varied by gender, the only exception being that the effect of adolescent-reported needs on use of youth social care was relatively larger for boys. Thus the increase of the severity of problems in girls may explain their increasing use of care as they grow older, but the effect of the problems that they themselves and their parents perceive is, per unit increase, no stronger than it is for boys. Regarding youth social care, this effect is even a bit weaker, which may also be interpreted as that the increase in emotional problems for girls, is not fully translated into use of care. This topic certainly deserves further study.

Needs were found to be associated with higher use of all types of care, but the further set of factors predicting higher use was small, concerned only predisposing factors, and varied somewhat by type of care. Lower family SES predicted higher use of general care but not of youth social care. In particular the latter contrasts with findings of previous studies that showed use of youth social care to be more likely in the case of socioeconomic disadvantage of the families involved. [14] One explanation might be that disadvantage is mediated by needs and by poor progress at school. Further predictors for use differ relatively little between youth social care and youth mental healthcare. The only real exception was parental divorce, which may e.g. lead to a need for temporary shelter of youth that is mostly provided by youth social care. This may be interpreted as another indication of a partial overlap of these two types of care in targeting the same groups of adolescents as far as it concerns care provided for behavioral and emotional problems. Differences may, however, be larger for other reasons of encounter, such as child protection in cases of youth social care.

It should be noted that a rather small set of factors explained a substantial part of the age/gender differences in use of care, with the strongest factor being the needs of adolescents as reported by their parents and further factors all concerning predisposing ones. Enabling factors, relating to the means that adolescents have to access care, did not contribute. Explanations for this might be that in most cases the parents decide what help will be sought for their child, or that access to care is good anyhow and thus does not require additional competencies. Moreover, it should be noted that we did not assess appropriateness of care, but only actual use of care. Determinants as found might thus theoretically have led to improper use.

Finally, the increase in use at older ages in girls was hardly explained by any factor. In contrast, in particular for boys differences became somewhat bigger after adjustment for both all factors and for only the needs and predisposing factors. The question remains how to explain this age-trend. Is it due to older adolescents and their parents being more effective in their seeking of care, and in passing the various filters of the care system as outlined by Goldberg and Huxley? [5], [7], [11] Or is it a period-effect instead of an age-effect, as in the Netherlands overall rates of use increased somewhat during the decade covered by this cohort? [13] Evidently, this issue requires further study.

### Strengths and Limitations

Our study has substantial strengths, in particular, its large sample, high response rate, longitudinal nature, and assessment of a wide range of types of care. Moreover, by using multilevel techniques in longitudinal data, we were able to control for individual factors that might otherwise yield spurious variations by age. However, some limitations should be noted as well. We could not assess all determinants at all ages, which might yield some overestimation of the relative effect of needs. In addition, differences by age as measured may also reflect changes in the care system over time. During the study period, 2001–2010, the design of the Dutch care system was relatively stable but use increased. [29] Second, we only assessed use, not its intensity. If such a differentiation were to be used, associations might have been stronger, but this would certainly require additional study. Moreover, use of care was measured based on reports, albeit parent-report and not self-report. Use of parent-report may have reinforced its validity, but may also have led to some underestimation of the prevalence rates at the fourth wave, age 19. At that age, not all parents may be fully aware of the use of care by their child. Because of the same reason, we did not assess parent-reported child behavioral and emotional problems at that age, causing the analyses on the importance of current need to cover only ages 10–16. A final limitation is that needs were measured as perceived needs, i.e. report on the CBCL and YSR, which may deviate from clinically assessed needs.

### Implications

General care was used much more frequently than specialized mental healthcare for adolescent behavioral and emotional problems. Maintaining and reinforcing expertise regarding this topic in primary and general medical care should therefore be prioritized. This type of care is much more accessible for troubled youth and might also be more cost-effective than specialized services.

A reinforcement of this expertise might also provide some counterbalance against the combined use of several types of care in a short period and the rather strong increase of use of specialized care at increasing ages by female adolescents. An explanation for that increase may be that general care does not adequately meet the specific needs of this group, or that it concerns improper use. This merits further study. Given the rather strong expansion of care for young people’s emotional and behavioral problems, ways of coping with these problems and adequately targeting those in greatest need deserve our utmost attention.
